# Multiplex cytokine analysis in *Mycobacterium avium* complex lung disease: relationship between CXCL10 and poor prognostic factors

**DOI:** 10.1186/s12879-019-3888-4

**Published:** 2019-03-18

**Authors:** Yuuki Bamba, Hiroshi Moro, Nobumasa Aoki, Takeshi Koizumi, Yasuyoshi Ohshima, Satoshi Watanabe, Takuro Sakagami, Toshiyuki Koya, Toshinori Takada, Toshiaki Kikuchi

**Affiliations:** Department of Respiratory Medicine and Infectious Diseases, Niigata University Graduate School of Medical and Dental Sciences, 757 Ichibancho, Asahimachi-dori, Chuo Ward, Niigata City, 951-8510 Japan

**Keywords:** Respiratory infections (nontuberculous), *Mycobacterium avium* complex (MAC), CXC motif ligand 10 (CXCL10), Cytokine panel

## Abstract

**Background:**

*Mycobacterium avium* complex lung disease (MAC-LD) can deteriorate rapidly to become fatal. Reported poor prognostic factors include radiographic findings, undernutrition, anemia and high inflammation test values. However, the association of these prognostic factors with the pathophysiology of the disease remains unknown. We aimed to clarify the pathophysiology of MAC-LD and develop a new biomarker that reflects the immune response to the disease.

**Methods:**

We performed the cytokine panel analyses of serum from patients with MAC-LD and compared each cytokine level with clinically negative prognostic factors (radiographic disease type, body mass index, albumin, C-reactive protein and hemoglobin) and high-resolution CT scores.

**Results:**

We analyzed 27 patients with MAC-LD, 6 with the fibrocavitary form and 21 with the nodular bronchiectatic form on high-resolution CT. Serum CXC motif ligand 10 (CXCL10) concentration was significantly elevated in patients with the fibrocavitary form (*p* = 0.008). CXCL10 levels correlated with body mass index (r = − 0.60, *p* = 0.0008), serum albumin concentration (r = − 0.45, *p* = 0.016) and high-resolution CT scores (r = 0.61, *p* = 0.0006). Among 14 patients initially untreated, antibiotic therapy was initiated for five during the study period. CXCL10 concentration was significantly higher in these patients (*p* = 0.046), and receiver operating characteristic analysis for CXCL10 concentration on treatment initiation produced an area under the curve of 0.844, with a sensitivity of 100%, specificity of 66.7%, and cut-off value of 366.5 pg/mL.

**Conclusion:**

We revealed cytokine profiles in patients with MAC-LD. Serum CXCL10 levels probably reflect the severity of MAC-LD. Our findings suggest that CXCL10 concentration may be a promising biomarker for managing treatment for patients with MAC disease of the lung.

**Electronic supplementary material:**

The online version of this article (10.1186/s12879-019-3888-4) contains supplementary material, which is available to authorized users.

## Background

Prevalence and incidence of nontuberculous mycobacterial lung disease (NTM-LD) have been increasing worldwide [1–3]. In Japan, the incidence rate of NTM-LD in 2014 was estimated to be 14.7 per 100,000 people [4]. The most common pathogen for NTM-LD is the *Mycobacterium avium* complex (MAC), which comprises of *M. avium* and *M. intracellulare* [1, 3, 5]. MAC lung disease (MAC-LD) is mainly classified into two forms: the nodular bronchiectatic type (NB) and the fibrocavitary type (FC) [5]. Although the 2007 American Thoracic Society and Infectious Diseases Society of America (ATS/IDSA) guidelines recommend a combination treatment of multiple drugs [5], antibiotic treatment has limited efficacy [6]. Because there is no consensus on the optimal timing to start antibiotic therapy and the treatment period, it would be beneficial to establish useful biomarkers to monitor therapeutic effectiveness and evaluate prognosis.

Although the prognosis for MAC-LD is generally good, disease progression varies between patients, with some experiencing rapid, fatal deterioration. A few reports have evaluated the severity of MAC-LD and its prognosis. We reported that profiling data of variable number tandem repeats of *M. avium* are associated with disease progression in MAC-LD [7]. Other studies have suggested that anti-glycopeptidolipid (GPL)-core antibodies reflect disease activity; for example, Kitada et al. reported that serum levels of anti-GPL-core antibodies in patients with MAC-LD were related to the severity of chest computed tomography (CT) findings and were decreased after chemotherapy [8, 9]. In addition, there have been several surveys of clinical prognostic factors, which indicated that radiographic findings, undernutrition, anemia and high inflammation test values may be negative prognostic factors for MAC-LD [10, 11].

Limited information is available about the cytokine networks involved in the pathogenesis of MAC-LD. Activation of T helper type 1 (Th1) cells and cytokine production play important roles against intracellular parasites such as mycobacteria, but their roles in MAC infection remain poorly understood. It has been reported that peripheral blood mononuclear cells from NTM-LD patients produced less Th1 cytokines [interferon-γ (IFN-γ), interleukin-12 (IL-12) and tumor necrosis factor-α (TNF-α)] than those from healthy controls [12, 13] and that Th17 immunity may be associated with susceptibility to NTM-LD [14]. In addition, a previous report suggested that lower CXC motif ligand 10 (CXCL10) levels were associated with therapeutic response [15]. Thus, differences in cytokine response may be implicated in the differences between patients in the progression of MAC-LD.

The aim of the present study was to elucidate the pathophysiology of MAC-LD and develop a new biomarker that reflects the immune response to the disease. To do this, we simultaneously measured the protein concentrations of 38 serum cytokines and growth factors (GFs) in patients with MAC-LD. We also investigated the association between these protein concentrations and the poor prognostic factors described above.

## Methods

### Study design and laboratory data

We performed a cross-sectional study in consecutive patients with MAC-LD who attended Niigata University Medical and Dental Hospital between April 2013 and March 2016 and fulfilled the 2007 ATS/IDSA diagnostic criteria [5]. Initiation of antimicrobial treatment was decided at the discretion of the attending physician. COBAS TaqMan MTB/MAI (Roche Diagnostics, Tokyo, Japan) was used for identifying of Mycobacterium species. Mycobacteria other than MAC and tuberculosis were identified by DNA–DNA hybridization method.

Clinical data were collected from electronic medical records, including age, BMI, medical history with medication and laboratory test results. Additionally, we measured data on nutrition (albumin and pre-albumin), anemia [blood cell counts and hemoglobin (Hb)], body iron status (serum iron, unsaturated iron binding capacity, total iron binding capacity and transferrin saturation) and inflammation [C-reactive protein (CRP), serum amyloid A and erythrocyte sedimentation rates (ESR)]. Patients who were not receiving treatment during registration were investigated to determine treatment was initiated within the study period. When the attending physician judged that disease was progressive, patients received treatment for MAC.

### Radiographic findings

Radiographic findings were established on the basis of the chest HRCT scan and divided into two groups—NB and FC—which were defined according to the ATS guidelines [5]. The NB form was defined if HRCT scanning showed multiple nodules and bronchiectasis, and the FC form based on apical fibrocavitary lesions. When the patients with multiple nodules and bronchiectasis in HRCT had apical fibrocavitary lesions, they were grouped into FC. As described in a previous report [16], radiographic findings were evaluated for the presence, distribution, and extent of the following signs: (1) cavities, (2) consolidation (panlobular and polylobular consolidation), (3) bronchiectasis, (4) fibrosis, (5) ground glass opacity, (6) miliary nodules (1–2 mm), (7) nodules (2–10 mm) and (8) bronchial wall thickening. Based on the HRCT findings, the lungs were divided into six zones: upper, middle and lower zones within the right and left lungs each. The HRCT scores were based on the percentage of lung parenchyma that showed evidence of each recorded abnormality: (1) involvement of < 25% of the image, (2) 25–50%, (3) 50–75%, and (4) > 75%. The total HRCT scores composed the sum of the eight parameter scores in the six zones. We defined the cavity score and the NB score as follows [16]. The cavity score was the sum of each cavity score (0–4) defined by the extent of the cavitary lesion for each zone (upper, middle and lower) within the right and left lungs. The NB score was the total number of parameters of the four parameters (bronchiectasis, miliary nodules, nodules, and bronchial thickening) in the six zones. These scores were calculated by reviews of the HRCT scans by two physicians who specialized in respiratory medicine (Y.B. and H.M.).

### Serum cytokine/growth factor protein level measurement

A panel of 38 cytokines was analyzed in the serum samples of all participants with MAC-LD using the Milliplex Map Human Cytokine/Chemokine Kit (Merck Millipore, Darmstadt, Germany), according to procedures previously described [17]. We analyzed the association among each cytokine level, clinically negative prognostic factors (radiographic disease type, BMI, albumin, CRP, and Hb) and HRCT scores. Similarly, we examined relationships between each cytokine concentration and the level of GPL core antibodies.

### Statistical analysis

We used the Shapiro–Wilk test to determine whether the values of the parameters were normally distributed normally. Data that were not normally distributed were reported as medians and interquartile ranges (median [25, 75%]). We performed parametric (Student’s *t* test) or nonparametric analysis (Wilcoxon rank sum test) for continuous and ordinal variables as appropriate. For categorical variables, we used Fisher’s exact test. Correlations between cytokines and clinical data were analyzed using the Spearman’s rank correlation test. To examine the association between cytokine concentration and the commencement of antibiotic treatment, we produced an receiver operating characteristic (ROC) curve after a univariate logistic regression model. *P* < 0.05 was considered statistically significant. All data were analyzed using JMP® 13 (SAS Institute Inc., NC, USA).

## Results

### Differences in characteristics between the FC and NB forms

Background characteristics of the 27 participants are summarized in Table 1. None were infected with HIV. The FC form was observed in six participants (22%), who had lower BMI and serum albumin and higher CRP and ESR than those with the NB form. Of the 21 participants with the NB form, 14 had not been treated at baseline. The HRCT scores were significantly higher in the FC form group (*p* < 0.05).

**Table 1:** Differences in characteristics between fibrocavitary and nodular bronchiectatic types (N = 27)

Characteristics	Radiographic Features		*P-value
	FC	NB	
Number (%)	6 (22)	21 (78)	
Age (y)	75 (61–78)	71 (64–78)	NS
Men (%)	2 (33)	3 (14)	NS
BMI (kg/m^2^)	16.5 ± 2.7	19.5 ± 2.5	< 0.05
Anemia (%)	3 (50)	7 (33)	NS
Iron deficiency (%)	2 (33)	3 (14)	NS
Undernutrition (%)	5 (83)	8 (38)	NS
With immunosuppressive drug (%)	0 (0)	7 (29)	NS
Untreated at baseline (%)	1 (17)	14 (67)	NS
Causative organism (%)			
*Mycobacterium. avium*	5 (83)	16 (76)	
*Mycobacterium*. *intracellulare*	1 (17)	3 (14)	
*M. avium* and *M. intracellulare*	0 (0)	2 (10)	
Lymphocytes (/μL)	1095 ± 307	1329 ± 417	NS
Red blood cells (× 10^4^/μL)	422 ± 39	418 ± 52	NS
Hb (g/dL)	12.5 ± 1.2	12.7 ± 1.5	NS
Albumin (g/dL)	3.1 (3.0–3.9)	4.0 (3.7–4.1)	< 0.01
Pre-albumin (mg/dL)	14.7 ± 10.0	21.9 ± 3.9	NS
Fe (μg/dL)	49 (19–105)	84 (49–109)	NS
UIBC (μg/dL)	192 ± 34	184 ± 70	NS
TIBC (μg/dL)	251 ± 37	265 ± 64	NS
TSAT (%)	22.6 ± 14.8	32.3 ± 17.2	NS
Ferritin (ng/mL)	74 (23–159)	48 (19–107)	NS
CRP (mg/dL)	4.6 (0.2–6.9)	0.1 (0.1–1.2)	< 0.05
SAA (mg/dL)	241.5 (6.3–726.2)	3.7 (2.4–28.7)	NS
ESR (mm/hr)	70 (31–96)	19 (750)	< 0.05
Anti-GPL core antibody positive (%)	5 (83)	11 (52)	NS
HRCT scores	25 (12–31)	9 (6–15)	< 0.05

Data are presented as number (%), mean ± standard deviation, or median (interquartile range)

Abbreviations, *FC* fibrocavitary type, *NB* nodular bronchiectatic type, *BMI* body mass index, *Hb* hemoglobin, *UIBC* unsaturated iron binding capacity, *TIBC* total iron binding capacity, *TSAT* transferrin saturation, *CRP* C-reactive protein, *SAA* serum amyloid A, *ESR* erythrocyte sedimentation rate, *GPL* glycopeptidolipid, *HRCT* high-resolution computed tomography

Definitions: Anemia: Hb < 12 g/dL in men, < 11 g/dL in women; Iron deficiency: TSAT < 20% and ferritin < 80 μg/mL; Undernutrition: albumin < 3 .5g/dL or pre-albumin < 22 mg/dL; Anti-GPL-core antibody-positive: > 0.7 U/mL

*P-values were calculated in relation to two radiographic features. Fisher’s exact test was used for categorical variables, and Student’s t-test or the Wilcoxon rank sum test for continuous variables. Welch’s t-test was used for pre-albumin. NS, not significant

### Comparison of cytokine/GF levels between the FC and NB forms

Concentrations of 38 cytokines/GFs were evaluated and compared between the two disease forms (Table 2). To ensure accurate comparison, we excluded certain items when > 50% cases were below or over the limit of detection (LOD). For enrolled cytokines/GFs, we replaced the values more or less than LOD with those of LOD and analyzed with the values using nonparametric analysis or Fisher’s exact test. We found five cytokines significantly higher in the FC group: G-CSF, IL-1RA, IL-10, CXCL10, and sCD40L.

**Table 2:** Serum cytokine profiles in two disease types of *Mycobacterium avium* complex lung disease

Cytokine/GF protein (pg/mL)			Radiographic Features		*P-value
	min LOD	max LOD	FC (*N* = 6)	NS (*N* = 21)	
Proinflamatory					
G-CSF	16	10,000	46.6 (32.4–68.4)	N/A	< 0.05
GM-CSF	0.64	10,000	11.4 (1.8–50.1)	3.5 (0.64–11.7)	NS
IFN-γ	3.2	10,000	12.8 (6.2–37.6)	3.9 (3.2–29.7)	NS
IL-1β	0.64	10,000	3.0 (0.64–20.6)	N/A	NS
IL-6	0.64	2000	38.6 (18.1–225.3)	21.7 (3.8–87.6)	NS
IL-7	0.64	2000	13.1 (6.3–32.5)	6.5 (0.64–9.0)	NS
IL-17A	0.64	10,000	3.1 (1.2–9.0)	1.7 (0.3–15.6)	NS
sCD40L	0.64	10,000	N/A	5278 (3960–6276)	< 0.05
TNF-α	0.64	10,000	50.6 (19.3–131.3)	45.2 (25.6–73.5)	NS
Chemokine					
Eotaxin	3.2	10,000	122 (101–134)	134 (112–168)	NS
GRO	16	10,000	1285 (1137–1784)	896 (771–1567)	NS
IL-8	0.64	10,000	361 (143–906)	230 (104–650)	NS
CXCL10	3.2	10,000	979 (757–1496)	451 (268–556)	< 0.05
CCL2	3.2	10,000	600 ± 120	600 ± 183	NS
CCL3	16	2000	345 (14–610)	216 (124–505)	NS
CCL4	16	10,000	191 (37–415)	173 (93–373)	NS
CCL22	16	10,000	863 ± 163	657 ± 309	NS
Anti-inflammatory					
IL-1RA	3.2	10,000	73.9 (31.3–136.5)	18.7 (3.2–29.4)	< 0.005
IL-5	0.64	10,000	1.9 (0.64–5.9)	N/A	NS
IL-10	0.64	10,000	3.5 (1.4–14.7)	N/A	< 0.05
Growth factor					
EGF	16	10,000	294 ± 66	247 ± 117	NS
FGF-2	16	10,000	94.3 (48.9–150.9)	55.0 (16.3–82.3)	NS
Flt-3 L	3.2	10,000	3.2 (3.2–21.7)	12.1 (3.2–27.4)	NS
TGF-α	0.64	2000	12.0 (10.3–18.9)	6.6 (4.6–13.0)	NS
VEGF	80	10,000	531 ± 318	300 ± 234	NS

Data are presented as mean ± standard deviation, or median (interquartile range). Items with more than half cases below or over the LOD were excluded

Abbreviations, *GF* growth factor, *LOD* limit of detection, *FC* fibrocavitary type, *NB* nodular bronchiectatic type, *EGF* epidermal growth factor, *FGF* fibroblast growth factor, *Flt-3 L* FMS-like tyrosine kinase 3 ligand, *G-CSF* granulocyte colony-stimulating factor, *GM-CSF* granulocyte macrophage-CSF, *GRO* growth-related oncogene, *IFN-γ* interferon gamma, *IL* interleukin, *IL-1RA* IL-1 receptor antagonist, *CXCL10* CXC motif ligand 10, *CCL* CC motif ligand, *sCD40L* soluble CD40 ligand, *TGF* transforming growth factor, *TNF* tumor necrosis factor, *VEGF* vascular endothelial growth factor, *N/A* not available (median was below or over LOD)

*P-values were calculated in relation to two radiographic features. Student’s t-test or the Wilcoxon rank sum test was used. For enrolled cytokines/growth factors, we replaced the values less than LOD with LOD and analyzed with nonparametric analysis or Fisher’s exact test with cut-off as LOD value. NS, not significant

### Cytokine/GF levels and negative prognosis factor of MAC-LD

We compared these five cytokines/GFs concentrations between the patients with and without the negative clinical prognosis factors for MAC-LD (low BMI, low albumin, high CRP and anemia) [10] (Table 3). Concentration of IL-1RA was statistically different only between low and high albumin levels with marginal significance (< 0.05), but it showed no significant difference between low and high BMI, low and high CRP, or anemic and non-anemic. As for CXCL10, it had highly significant difference in two statistical comparisons, i.e., between low and high BMI as well as low and high albumin levels. Thus, we suspected that CXCL10 plays an important role in the progression of the disease.

**Table 3:** Comparison of concentrations of five cytokines between participants with and without poor prognostic factors (N = 27)

Cytokine/GF protein (pg/mL)	Poor prognostic factors	Low	High	^*^ *P-value*
G-CSF	BMI	19.6 (16–42.5)	N/A	NS
min LOD: 16	Alb	54.8 (25.8–70.1)	N/A	NS
max LOD: 10000	CRP	N/A	21.5 (16–66.7)	NS
	Hb	35.6 (16–125.8)	N/A	NS
IL-1RA	BMI	28.2 (10.5–63.9)	17.4 (3.2–24.9)	NS
min LOD: 3.2	Alb	43.2 (22.5–144.4)	19.6 (3.2–30.1)	< 0.05
max LOD 10000	CRP	15.5 (3.2–46.2)	29.1 (17.3–43.2)	NS
	Hb	29.1 (12.9–38.0)	19.6 (3.2–57.5)	NS
IL-10	BMI	1.0 (0.64–4.2)	1.3 (0.64–2.9)	NS
min LOD: 0.64	Alb	2.0 (0.9–18.6)	0.7 (0.64–3.6)	NS
max LOD: 10000	CRP	N/A	1.3 (0.64–4.0)	NS
	Hb	1.3 (0.64–18.6)	0.7 (0.64–2.6)	NS
CXCL10	BMI	953 (429–1568)	359 (226–456)	0.005
min LOD: 3.2	Alb	1030 (774–1816)	449 (274–554)	< 0.005
max LOD: 10000	CRP	445 (263–592)	551 (367–1030)	NS
	Hb	622 (286–1103)	455 (339–593)	NS
sCD40L	BMI	5941 (4235–10,000)	5278 (2808–7197)	NS
min LOD: 0.64	Alb	7611 (4739–10,000)	5317 (3973–8380)	NS
max LOD: 10000	CRP	4866 (3948–6210)	6323 (4300–10,000)	NS
	Hb	5845 (3812–10,000)	5678 (3973–9481)	NS

Data are presented as median (interquartile range). Items with more than half cases below or over the LOD were excluded

Abbreviations, *GF* growth factor, *LOD* limit of detection, *G-CSF* granulocyte colony-stimulating factor, *BMI* body mass index, *Alb* albumin, *CRP* C-reactive protein, *Hb* hemoglobin, *IL-1RA* interleukin-1 receptor antagonist, *CXCL10* CXC motif ligand 10, *sCD40L* soluble CD40 ligand, *N/A* not available (median was below or over LOD)

Definitions: low body mass index: < 20 kg/m^2^; low albumin: < 3.5 g/dL; high C-reactive protein: > 0.3 mg/dL; Anemia: Hb < 12.0 g/dL in males and < 11.0 g/dL in females

^*^P-values were calculated using Wilcoxon rank sum test. If more than half of one group was lower LOD, we used Fisher’s exact test with cut-off as LOD value. NS, not significant

**Fig. 1:**
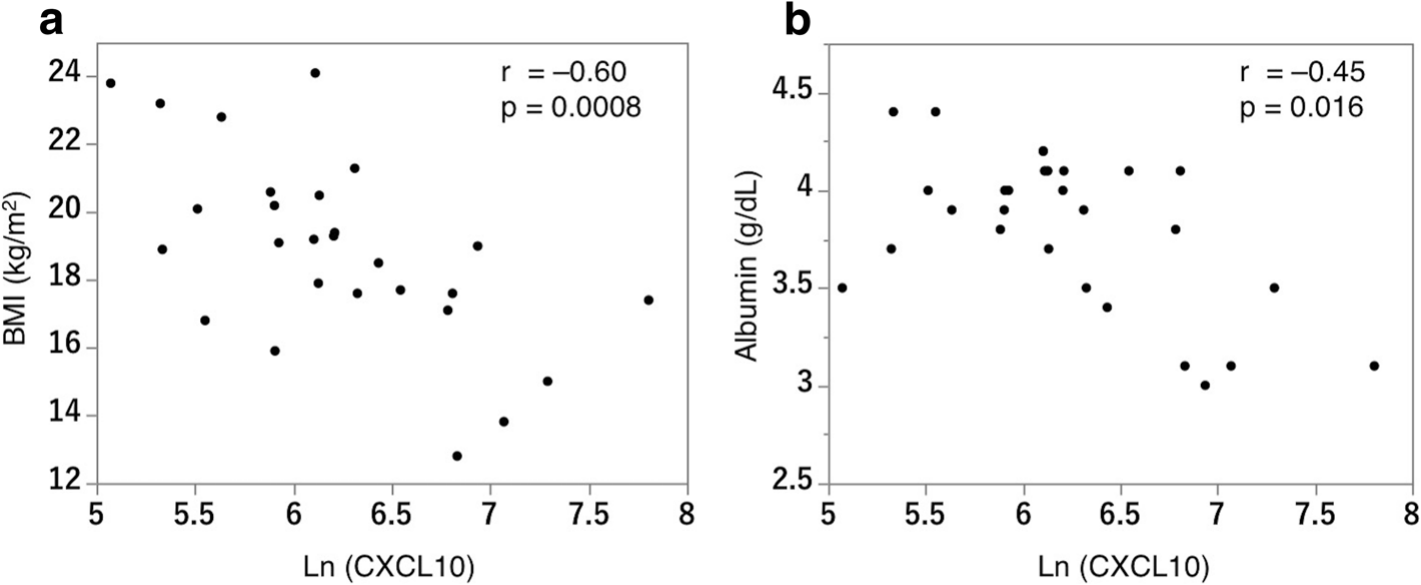
Correlations between CXCL10 concentration and body mass index (BMI) (**a**) and between CXCL10 concentration and serum albumin (**b**) in patients with MAC-LD (*N* = 27). The concentration of CXCL10 presented negative correlations with BMI (r = − 0.60, *p* = 0.0008) and albumin (r = − 0.45, *p* = 0.016). Spearman’s rank correlation coefficient was used to examine the relationship between CXCL10 and BMI and serum albumin. Ln: natural logarithm

We then analyzed correlations between CXCL10 and the poor prognostic factors of low BMI and albumin levels. We found moderate, significantly negative correlations between CXCL10 concentrations and BMI (*r* = − 0.60, *p* = 0.0008) and between CXCL10 and serum albumin levels (r = − 0.45, *p* = 0.016) (Fig. 1). In the NB form group, while BMI was lower than that observed in the FC form, CXCL10 and BMI were also significantly correlated (r = − 0.49, *p* = 0.024; Additional file 1: Figure S1).

### Relationship between CXCL10 concentration and the HRCT scores

**Fig. 2:**
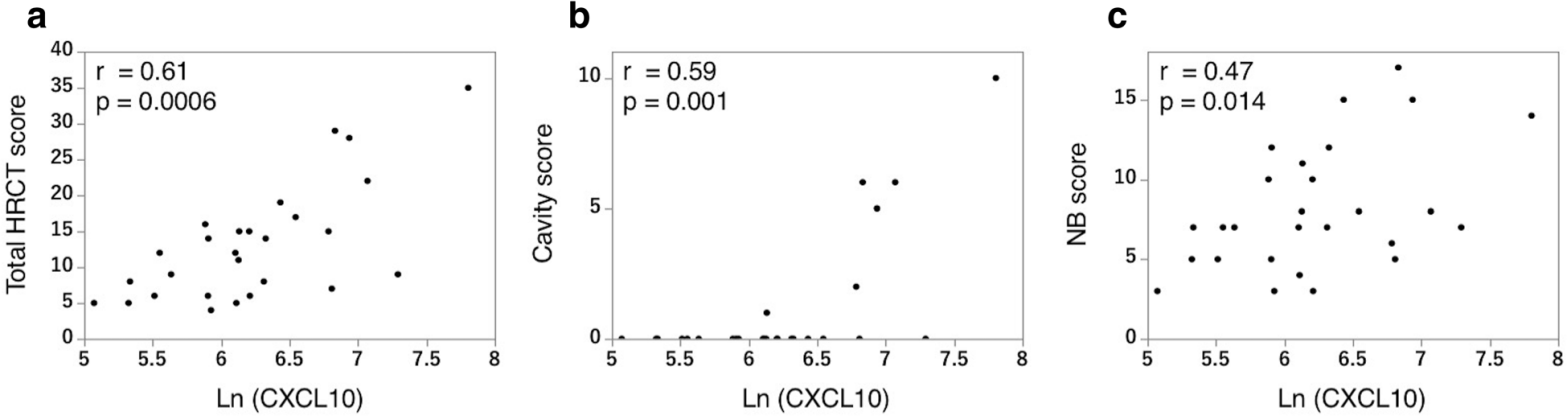
Correlations between CXCL10 concentration and high-resolution computed tomography (HRCT) scores. We evaluated HRCT findings for the presence, distribution and extent of the eight signs: cavities, consolidation, bronchiectasis, fibrosis, ground glass opacity, miliary nodules, nodules and bronchial wall thickening. The total HRCT score was the sum of the scores for the whole lung fields. The nodular bronchiectatic (NB) score was the total of four parameters (bronchiectasis, miliary nodules, nodules and bronchial wall thickening). The CXCL10 concentration positively correlated with the total HRCT score (**a**), and especially with the cavity score (**b**). It also showed a moderate positive association with the NB score (**c**). Spearman’s rank correlation coefficient was used to examine the relationship between CXCL10 and the HRCT scan scores. Ln: natural logarithm

We analyzed the relationship between CXCL10 concentration and the HRCT scores (Fig. 2). The CXCL10 concentration positively correlated with the HRCT score (r = 0.61, *p* = 0.0006), with the relationship stronger for the cavity score (r = 0.59, *p* = 0.001 for the cavity score and r = 0.47, *p* = 0.014 for the NB score). Regarding the levels of the other cytokines, IL-1RA also showed low correlation with the total HRCT scores (Additional file 1: Figure S2).

### Usefulness of serum CXCL10 concentration as a predictive marker for the progression of MAC-LD

**Fig. 3:**
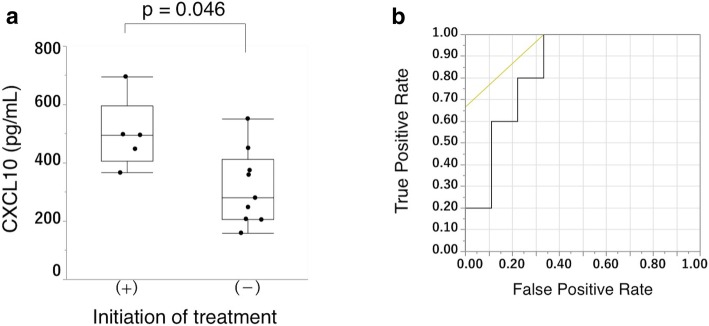
Comparison of CXCL10 concentrations between the patients who initiated treatment and those who did not. (**a**) Five of the fifteen patients previously untreated were commenced on antimicrobial therapies during the study period. After excluding a patient aged over 80 years who was ineligible for antibiotic treatment because of age, CXCL10 was significantly higher in the patients who commenced antimicrobial treatment (*p* = 0.046). (**b**) Receiver operating characteristic curve of the CXCL10 concentration and treatment commencement for 14 patients. Sensitivity and specificity calculated were 100 and 66.7%, respectively, when the CXCL10 cut-off was defined as 366.5 pg/mL, with area under the curve of 0.844

Among the 15 initially untreated participants, antibiotic therapy was commenced for five during the study period. The reason for the initiation of treatment was the progression of symptoms or image findings judged by the attending physicians. An 80-year-old patient was excluded from the analysis because antibiotic treatment was not prescribed owing to his high age. Additional file 1: Table S1 summarizes the characteristics of the 14 untreated patients. We compared CXCL10 concentrations between the untreated and those administered antibiotic treatment against MAC during the study period CXCL10 concentrations tended to be higher in the treated group than the other, but this difference was not significant (Additional file 1: Figure S3). However, excluding the elderly patient, CXCL10 concentration was found to be significantly higher in the treated group (Fig. 3a). ROC analysis for CXCL10 on treatment commencement for the 14 patients showed an area under the curve of 0.844, with sensitivity of 100% and specificity of 66.7% for a cut-off value of 366.5 pg/mL for the CXCL10 concentration (Fig. 3b).

## Discussion

The natural history of MAC-LD depends on the type of clinical disease: FC or NB [5]. A radiographic finding of FC is one of the negative prognostic factors for both all-cause and MAC-specific mortality. We measured 38 serum cytokine/GFs protein concentrations in 27 patients with MAC-LD and compared them between those with the FC and the NB forms. We found that CXCL10 concentrations were significantly higher in those with the FC form and that CXCL10 concentration correlated with other poor prognostic factors, the HRCT scores and disease activity.

MAC-LD typically presents as an apical fibrocavitary lung disease, FC form. If left untreated, this form of disease is gradually progressive, resulting in extensive cavitary lung destruction with increased mortality [10, 18, 19]. On the other hand, the NB form tends to have a much slower progression than the FC form, but it too may progress, leading to death [20]. Using a multivariate Cox proportional hazard model, Hayashi et al. found that a radiographic finding of FC or FC combined with NB was a negative prognostic factor for MAC-specific mortality, as were low BMI, presence of anemia and high CRP [10]. We investigated the relationship between these poor prognostic factors and serum cytokine concentrations. It is plausible that levels of many cytokines were low because they act locally and transiently rather than systemically and chronically. Two proinflammatory cytokines, G-CSF and sCD40L, and one chemokine, CXCL10, were significantly elevated in the FC group, whereas anti-inflammatory cytokines, IL-1RA and IL-10, were significantly elevated in the FC group. This finding might indicate that a strong immune response to MAC infection and accompanying inhibitory response are variable, particularly in the FC form.

Among the cytokines analyzed, CXCL10 levels were associated with low BMI, low albumin and high HRCT scores. CXCL10 expressed by antigen-presenting cells induces chemotaxis, apoptosis, cytostasis and angiostasis [21–23]. As well as in several autoimmune diseases [24] and in infections with various viruses [25–27], CXCL10 has been reported to be a useful biomarker for tuberculosis (TB) [28, 29]. Another report indicated that plasma CXCL10 levels in patients with MAC-LD were higher than in those healthy participants [15]. Our results show that CXCL10 is associated with CT findings and need for treatment of MAC-LD. Therefore, it increases against MAC infection and may be involved in the pathogenesis of the disease.

Although skin test reactivity to MAC is highly prevalent during young adulthood [30], pulmonary MAC disease is relatively rare, suggesting the host immune response plays an important part in eliminating the infecting microbes. Inhaled mycobacteria are taken up by macrophages, but they survive and proliferate within these vacuoles as an intracellular pathogen. Macrophages containing MAC produce cytokines to recruit lymphocytes and other macrophages. Of these cytokines, IL-12, TNF-α, and IFN-γ are of particular importance in the anti-mycobacterial immune response. IFN-γ and IL-12 form a positive feedback loop that is pivotal in the immune response to mycobacteria [31, 32]. The production of CXCL10 is driven by many signals, including IFN-γ, IFN-α/β, IL-2, and autocrine antigen-presenting cell-derived TNF-α and IL-1β in an autocrine manner [33–35]. We found a significant increase in the concentration of CXCL10 between the FC and NB subtypes, but not in IFN-α, IL-2, TNF-α, or IL-1β. Perhaps the poor increase of these inflammatory cytokines may contribute to the pathology of MAC disease [12, 13].

With MAC-LD, one of the major problems in clinical practice is when to start antibiotics treatment. If untreated, approximately half of patients with MAC-LD progress over the subsequent 2–10 years of follow-up [11, 18, 36, 37]. However, there is still no clear standard for the initiation of treatment, especially for patients with NB form. Risk factors for disease progression include low BMI, cavitary disease on chest CT, number of lung segments involved, older age, male sex, and the presence of comorbidities, as well as anemia, hypoalbuminemia and elevated CRP and/or ESR levels [11, 18, 36]. In the present study, we found statistically significant associations between CXCL10 levels and some of these risk factors. In addition, CXCL10 was significantly higher in the patients requiring treatment, as in a previous report [15]. Thus, CXCL10 could be a promising biomarker for determining the treatment strategy for the disease.

Anti-GPL-core IgA antibody is reported to be a convincing diagnostic marker for MAC-LD [38]. Some studies have suggested that this antibody may reflect the progression of MAC-LD to some extent [8, 9]. However, in the present study, we did not find a statistical difference in anti-GPL-core IgA antibody levels that supported this hypothesis. Instead, we found that CXCL10 concentration was strongly correlated with the poor prognostic factors and the HRCT scores, suggesting that the cytokine may reflect the disease activity with greater sensitivity than anti-GPL-core IgA antibody levels.

The current study had some limitations. First, it was a cross-sectional, single-center study. The possibility of unintentional selection bias could not be ruled out. In addition, because our institution is a university hospital, there may have been other biases in patient recruitment. Second, the number of participants was small, including only six cases with the FC form of MAC-LD, which may have resulted in underestimates of the differences in cytokine concentrations. The number of patients was insufficient to analyze the association between CXCL10 and the poor prognosis factors for each of the two disease form groups separately. For the same reason, we could not efficiently exclude several confounding factors. In addition, the standard of treatment introduction was unclear. Our findings need to be confirmed in a larger cohort from multiple institutions, preferably in a prospective study for a longer study period to establish CXCL10 as a biomarker for prediction of the prognosis of MAC-LD.

## Conclusion

In conclusion, our findings reveal the cytokine profile in MAC-LD and suggest that CXCL10 may be deeply involved in the pathogenesis and reflects the severity of the disease. Measurement of serum CXCL10 concentration may be useful for assessing disease activity and managing treatment for patients with MAC disease of the lung.

## Additional file

Additional file 1: Additional tables and figures to support the main article. (DOCX 2155 kb)

## Abbreviations

BMI: Body mass index
CRP: C-reactive protein
CXCL10: CXC motif ligand 10
FC: Fibrocavitary disease
Hb: hemoglobin
HRCT: High-resolution computed tomography
MAC-LD: *Mycobacterium avium* complex lung disease
NB: nodular bronchiectatic disease
