# Supplementary material for: Multiplex cytokine analysis in Mycobacterium avium complex lung disease: relationship between CXCL10 and poor prognostic factors
Source: BMC Infect Dis. 2019 Mar 18;19:263. doi: 10.1186/s12879-019-3888-4 (PMC6423821; doi:10.1186/s12879-019-3888-4)

**Additional Files**

**Table S1. Comparison of characteristics between the unstable group vs stable group.**

Characteristics Unstable Stable *P-value**

Number 5 9

Age (yr) 71 (69-75) 65 (50-76) NS

Men (%) 1 (20) 3 (22) NS

BMI (kg/m^2^) 19.2 ± 0.9 21.6 ± 2.0 <0.05

Hb (g/dL) 13.4 ± 0.8 12.9 ± 1.4 NS

Albumin (g/dL) 4.1 (3.9– 4.2) 3.9 (3.7– 4.1) NS

CRP (mg/dL) 0.3 (0.0– 1.3) 0.1 (0.0– 1.1) NS

HRCT scores 12 (6- 16) 6 (5- 9) NS

BMI: body mass index; Hb: hemoglobin; CRP: C-reactive protein. Data are presented as number (%), Data are presented as mean ± standard deviation, or median (interquartile range). * Student’s t-test or the Wilcoxon rank sum test was used for continuous variables. NS, not significant.

**Figure S1. Correlations between CXCL10 concentration and body mass index (BMI) in nodular bronchiectatic form group (N = 21).** The concentration of CXCL10 presented negative correlations with BMI (r = −0.49, *P* = 0.024). Spearman’s rank correlation coefficient was used to examine the relationship between CXCL10 and BMI. Ln: natural logarithm.

**
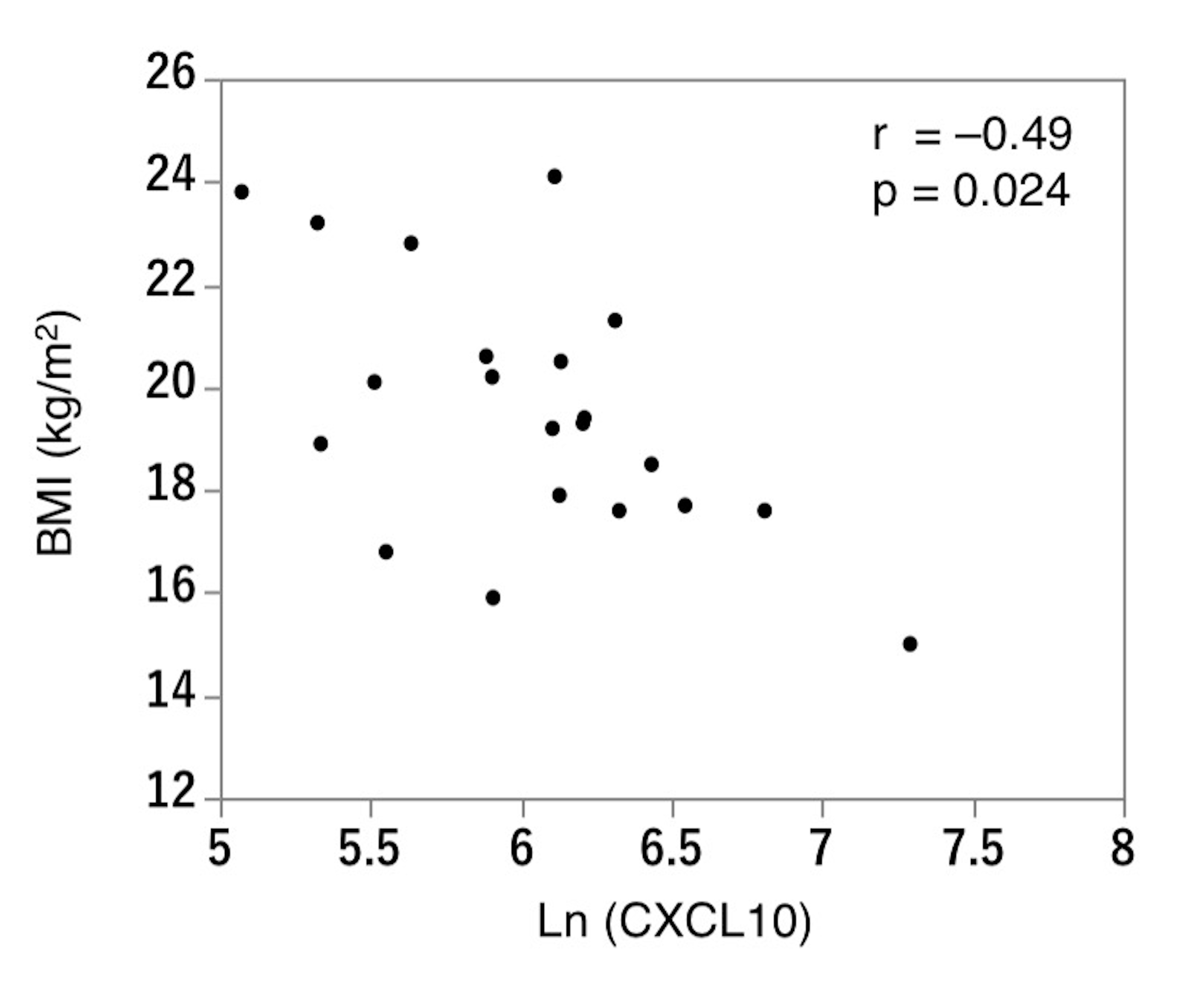
**

**Figure S2. Correlation between Interleukin-1 receptor antagonist (IL-1RA) and high-resolution CT scores.** IL-1RA levels was weakly correlated with the total high-resolution CT (HRCT) scores. Spearman’s rank correlation coefficient was used to examine the relationship between IL-1RA and HRCT score.

**
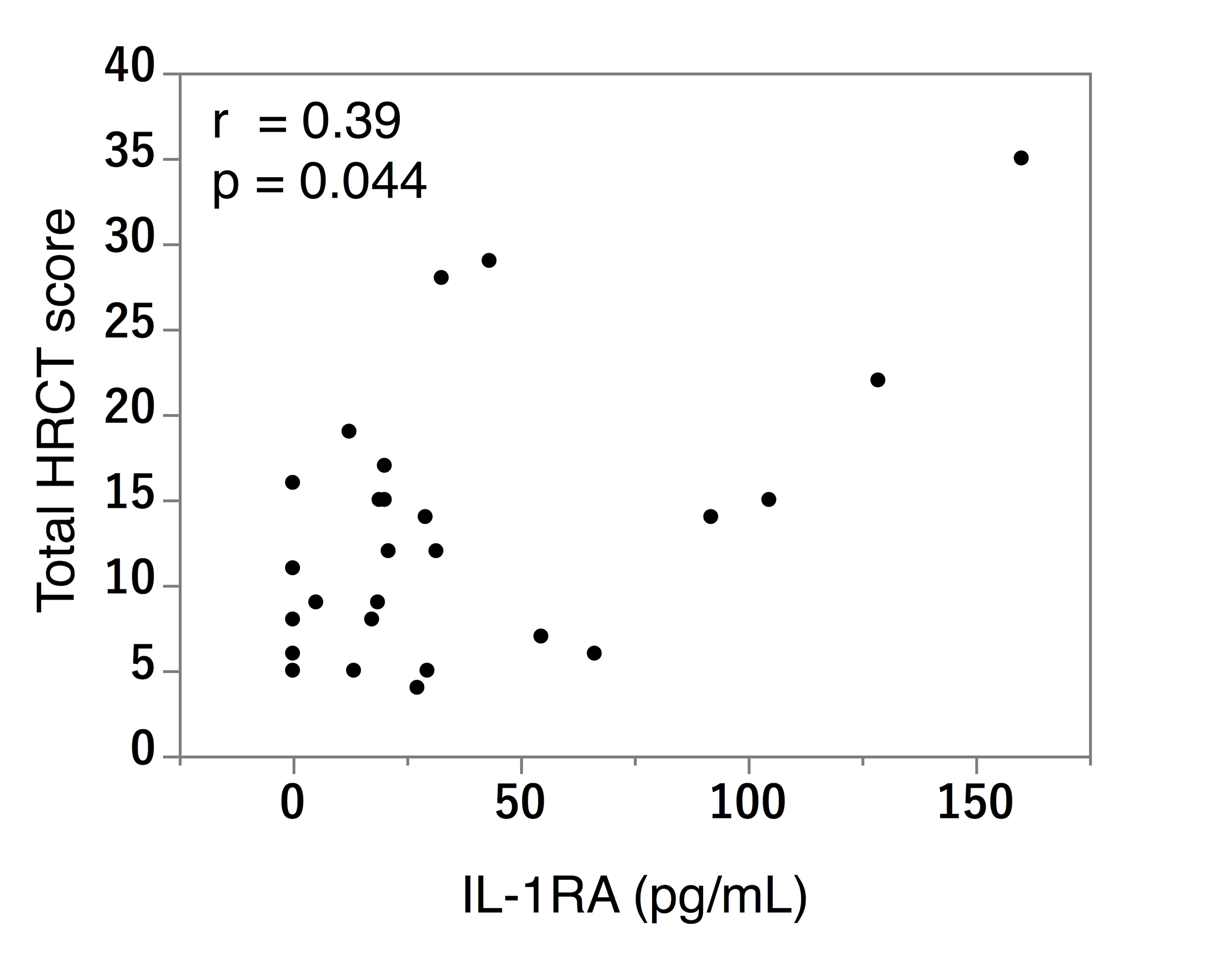
**

# **Figure S3. Comparison of CXCL10 concentrations between the patients subsequently treated and those not.** Five of the 15 patients previously untreated were commenced on antimicrobial therapies during the study period. The concentration of CXCL10 tended to be higher in the treated group, but there was no significant difference (*P* = 0.099).

##
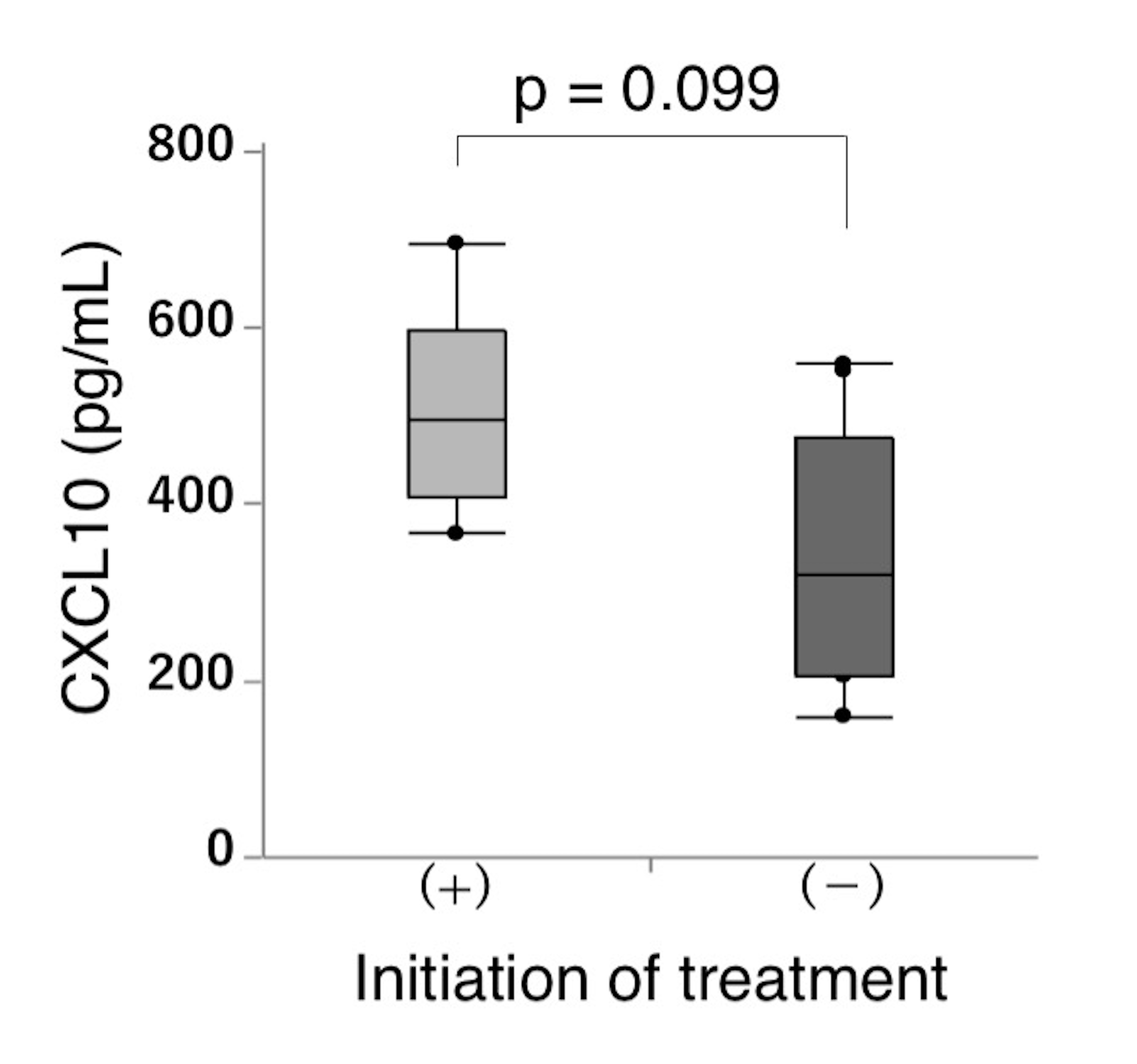

Supplement: Supplementary file 1 — Additional tables and figures to support the main article. (DOCX 2155 kb) [file 12879_2019_3888_MOESM1_ESM.docx]
